# Incidence and Risk Factors for De Novo Chronic Rhinosinusitis in Kidney and Liver Transplant Recipients

**DOI:** 10.1002/oto2.70200

**Published:** 2026-02-16

**Authors:** Bastien A. Valencia‐Sanchez, Hannah Daniel, Prishae Wilson, Natasha N. Najmi, Ryan Goodman, Hani M. Wadei, Denise Harnois, Angela M. Donaldson

**Affiliations:** ^1^ Department of Otolaryngology–Head and Neck Surgery Mayo Clinic Florida Jacksonville Florida USA; ^2^ Department of Transplant Medicine Mayo Clinic Florida Jacksonville Florida USA; ^3^ Department of Gastroenterology and Hepatology Mayo Clinic Florida Jacksonville Florida USA

**Keywords:** chronic rhinosinusitis, kidney transplant, liver transplant, incidence, risk factors

## Abstract

**Objective:**

To estimate the incidence of de novo chronic rhinosinusitis and identify associated risk factors in kidney and liver transplant recipients without pre‐existing sinonasal complaints.

**Study design:**

Retrospective cohort study.

**Setting:**

Multisite study across Mayo Clinic Enterprise locations in Arizona, Florida, Minnesota, and Wisconsin.

**Methods:**

Records of all patients who underwent kidney or liver transplantation between November 1, 2021, and November 1, 2022 were reviewed. Patients with documented sinonasal complaints prior to transplantation were excluded. Diagnoses were based on ICD‐10 codes assigned by board‐certified otolaryngologists, with follow‐up extending through November 2024. Demographic and clinical variables were collected, and multivariable logistic regression was used to identify predictors of de novo chronic rhinosinusitis.

**Results:**

Among 1459 transplant recipients (mean age 55.1 years, 59.8% male), the cumulative 2‐year incidence of de novo chronic rhinosinusitis was 2.4% (24/986) in kidney and 2.3% (11/473) in liver transplant recipients (*P* = .89). The overall incidence rate was 12.0 cases per 1000 person‐years, exceeding general population estimates. Transplant rejection (OR 3.2, 95% CI 2.5‐3.9, *P* < .001) and additional organ transplantation (OR 5.0, 95% CI 3.4‐6.5, *P* = .041) were independently associated with chronic rhinosinusitis development.

**Conclusion:**

Kidney and liver transplant recipients experience a higher incidence of de novo chronic rhinosinusitis compared to the general population. Transplant rejection and additional organ transplantation significantly increase this risk. Early recognition and management of chronic rhinosinusitis in this population may support improved postoperative outcomes.

Chronic rhinosinusitis (CRS) is a relatively common inflammatory condition associated with significant morbidity, decreased quality of life, and substantial healthcare costs.^1^ Concurrently, the number of solid organ transplant recipients has steadily increased over the past decades, representing a growing population with complex healthcare needs and considerable economic impact.^2^ Immunosuppressed individuals, including transplant recipients, are known to be at higher risk for both infectious and chronic inflammatory conditions such as CRS.^3^, ^4^ However, existing studies evaluating CRS in transplant populations have notable limitations.

Prior research has largely focused on prevalence rather than incidence, failing to distinguish between pre‐existing sinonasal disease and CRS that develops post‐transplantation.^5^, ^6^ Additionally, reported prevalence estimates vary widely, ranging from 0.6% to 30.4%, likely reflecting differences in study populations and diagnostic criteria.^5^, ^7^ Finally, past studies have combined heterogenous transplant populations (ie, both solid and nonsolid organ transplant recipients), despite important differences in comorbidities and immunosuppressive regimens that may influence CRS risk.^8^

Kidney and liver transplant recipients represent a particularly relevant study population for several reasons. Together, they account for over 80% of all solid organ transplants performed annually, according to Organ Procurement and Transplant Network data.^2^ This population has also been reported to have a higher risk of medically recalcitrant CRS compared to both the general population and other transplant recipients.^8^ Importantly, kidney and liver transplant recipients tend to receive relatively similar immunosuppressive medications, typically receiving a combination of calcineurin inhibitors, antimetabolites, and corticosteroids.^9^, ^10^ Despite this, the incidence of de novo disease, defined as new‐onset disease in patients without sinonasal symptoms prior to transplantation, remains poorly characterized in this population. Additionally, specific risk factors for CRS development in kidney and liver transplant recipients are not well understood.

This study aims to estimate the incidence of de novo CRS in kidney and liver transplant recipients. A secondary aim is to identify potential clinical risk factors associated with its development.

## Methods

### Data Source

This multisite retrospective cohort study included patients who underwent kidney or liver transplantation at the Mayo Clinic Enterprise between November 1, 2021, and November 1, 2022. The study was deemed exempt from IRB review (ID 22‐000831). The initial cohort was identified using the Mayo Data Explorer and included patients from Mayo Clinic sites in Arizona, Florida, Minnesota, and Wisconsin.

### Study Population

Eligible patients were those who underwent kidney or liver transplantation and follow‐up within the Mayo Clinic Enterprise during the study period. Patients with documented sinonasal complaints prior to transplantation, as evaluated in our tertiary rhinology clinic, were excluded from the analysis.

CRS diagnoses were identified using ICD‐10 codes assigned by board‐certified otolaryngologists. Follow‐up for the development of the primary outcome (CRS) extended through November 2024.

A 2‐year follow‐up period was selected to capture the first year posttransplant, during which time immune‐related complications such as acute transplant rejection, graft‐versus‐host disease, opportunistic infections, and posttransplant lymphoproliferative disorder are most likely to occur.^9^, ^11^ This timeframe is further supported by prior studies in hematopoietic stem cell transplant recipients reporting a median time to rhinosinusitis diagnosis of approximately 200 days.^12^

Demographic data, relevant comorbidities (asthma and allergies), transplant‐related variables (organ transplanted, transplant rejection, additional organ transplant, maintenance immunosuppressive regimen), and need for endoscopic sinus surgery (ESS) to manage CRS were extracted from the medical record.

### Statistical Analysis

Statistical analyses were performed using IBM SPSS Statistics for Windows, version 28.0 (IBM Corp.). Continuous variables were reported as means ± standard deviations (SD). Categorical variables were summarized as frequencies and percentages. Both cumulative incidence (over a 2‐year period) and incidence rates (per 1000 person‐years [PY]) were reported.

Group comparisons for continuous variables were performed using the independent samples *t*‐test or the Mann‐Whitney *U* test, contingent on data normality and equality of variances. Normality was assessed using the Shapiro‐Wilk test, and equality of variances using Levene's test. Categorical variables were compared using Pearson's chi‐square test or Fisher's exact test, contingent on cell counts. Multivariable logistic regression was used to identify predictors of de novo CRS, adjusting for relevant comorbidities. A *P* < .05 was considered statistically significant.

## Results

A total of 1459 patients met inclusion criteria and were included in the final analysis. The mean age at the time of organ transplantation was 55.1 ± 13.8 years with a male to female ratio of 1.5:1. Table 1 shows the baseline characteristics of the study cohort. Table 2 shows baseline characteristics stratified by transplanted organs. A total of 35 (2.4%) patients developed de novo CRS. Thirty‐four patients had chronic rhinosinusitis without nasal polyps (CRSsNP) and 1 patient had chronic rhinosinusitis with nasal polyps (CRSwNP).

**Table 1:** Baseline Characteristics of the Study Cohort (N = 1459)

Characteristic	Value
Age at transplant, mean ± SD (years)	55.1 ± 13.8
Male sex, n (%)	873 (59.8%)
Race/Ethnicity	—
White (Non‐Hispanic/Latino)	887 (60.8%)
Hispanic or Latino	236 (16.2%)
African American	173 (11.9%)
Asian	114 (7.8%)
American Indian/Alaskan Native	30 (2.1%)
Native Hawaiian/Pacific Islander	4 (0.3%)
Unknown	15 (0.9%)
Follow‐up duration (years)	2.0
Allergies	636 (43.6%)
Asthma	160 (11.0%)
Transplant rejection	399 (27.3%)
Additional organ transplant	21 (1.4%)

Abbreviations: N, study population; n, sample size; SD, standard deviation.

**Table 2:** Baseline Characteristics Stratified by Transplanted Organ

Characteristic	Kidney (n = 986)	Liver (n = 473)
Age at transplant, mean ± SD (years)	54.9 ± 14.4	55.5 ± 12.4
Male sex, n (%)	591 (59.9%)	282 (59.6%)
Race/ethnicity	—	—
White (Non‐Hispanic/Latino)	531 (53.9%)	356 (75.3%)
Hispanic or Latino	161 (16.3%)	75 (15.9%)
African American	163 (16.5%)	10 (2.1%)
Asian	90 (9.1%)	24 (5.1%)
American Indian/Alaskan Native	26 (2.6%)	4 (0.8%)
Native Hawaiian/Pacific Islander	4 (0.4%)	0 (0%)
Unknown	11 (1.2%)	4 (0.8%)
Allergies	413 (41.9%)	223 (47.1%)
Asthma	105 (10.6%)	55 (11.6%)
Transplant rejection	235 (23.8%)	164 (34.7%)
Additional organ transplant	20 (2.0%)	1 (0.2%)

Abbreviations: n, sample size; SD, standard deviation.

The cumulative incidence during the 2‐year follow‐up period was 2.4% (24/986) for kidney recipients and 2.3% (11/473) for liver recipients. There was no significant difference in the incidence of de novo CRS among kidney and liver recipients (*P* = .89). The overall incidence rate was 12.2 cases per 1000 PY for kidney recipients and 11.6 cases per 1000 PY for liver recipients.

On univariate analysis, transplant rejection (*P* < .001) and additional organ transplantation (*P* = .032) were significantly associated with de novo CRS.

Multivariable logistic regression controlling for relevant comorbidities (ie, asthma and allergies) identified transplant rejection (OR = 3.2, 95% CI 2.5, 3.9, *P* < .001) and additional organ transplant (OR = 5.0, 95% CI 3.4, 6.5, *P* = .041) as significant predictors of de novo CRS development.

ESS was required for symptom control in 17.1% (6/35) of patients. The rate of ESS was similar between kidney (4/24, 16.7%) and liver (2/11, 18.2%) recipients (*P* = .962).

## Discussion

**Figure 1:**
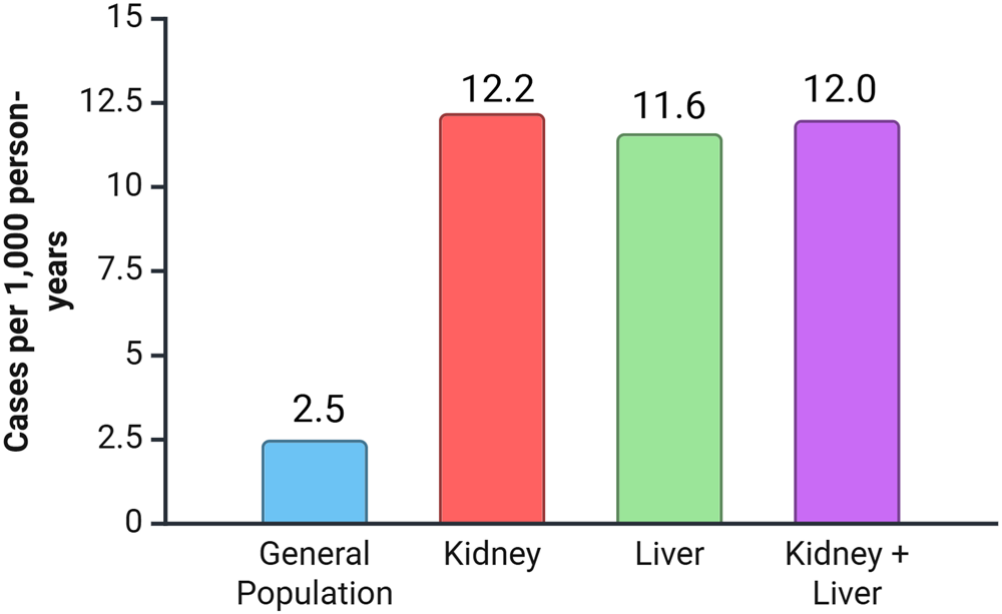
Incidence of de novo chronic rhinosinusitis description: incidence per 1000 person‐years in the general population versus transplant recipients, showing substantially higher rates among transplant recipients.

In this multisite retrospective cohort study of kidney and liver transplant recipients, we found that the cumulative two‐year incidence of de novo CRS was 2.3% in kidney transplant recipients and 2.4% in liver transplant recipients. These incidence rates are markedly higher than those reported in the general population (Figure 1). A recent global systematic review estimated the pooled annual incidence of CRS at 0.73%,^13^ while a population‐based study from Alberta, Canada, reported an incidence of just 0.25% (2.5 per 1000 population).^14^ Prior studies in transplant populations have primarily reported prevalence estimates rather than incidence. For example, one large retrospective study found a posttransplant CRS prevalence of 0.66% among 4562 solid organ transplant recipients,^6^ while another study of 1503 kidney transplant recipients reported a posttransplant prevalence of 0.6%.^7^ This last study excluded any patients with abnormal computed tomography (CT) findings, such as opacity within the ostiomeatal complex or sinuses, even if they were asymptomatic, likely underestimating the true post‐kidney transplant CRS incidence.^7^ One retrospective reported a CRS rate of 2.6% (26/996 patients) in a Korean cohort of liver transplant recipients. Although this rate is similar to that observed in our study, the authors included patients who met CRS diagnostic criteria during the year prior to transplantation, rather than identifying de novo cases following transplant.^15^

In our study, immunosuppressive therapy was relatively homogeneous as all patients had a maintenance immunosuppressive regimen consisting of a combination of a calcineurin inhibitor (tacrolimus or cyclosporine), an antimetabolite (mycophenolate mofetil), and a glucocorticoid (prednisone). The heightened susceptibility to CRS in our study may reflect the effects of chronic immunosuppression and immune dysregulation following transplantation. Immunosuppressive therapies diminish the capacity to clear pathogens and repair epithelial injury by impairing mucosal barrier function and inhibiting both innate and adaptive immune responses.^16^ Growing evidence supports the concept that CRS arises from a multifactorial disruption of the sinonasal mucosa. Genetic predisposition, allergen exposure, microbial infections, environmental pollutants, and tissue hypoxia have all been described as potential triggers of epithelial barrier dysfunction.^17^, ^18^ Once the epithelial barrier is compromised, not only is microbial and allergen penetration facilitated, but epithelial‐derived cytokines are released, activating downstream molecular pathways that perpetuate a pro‐inflammatory cycle. Persistent epithelial injury may also initiate a maladaptive repair response that reactivates the epithelial–mesenchymal transition (EMT), a cellular process that contributes to tissue remodeling and disease progression.^18^ In susceptible individuals, such as transplant recipients with impaired epithelial repair capacity, these converging factors may be further amplified. The pathogenesis of CRS in transplant patients may be further influenced by the disruption of the sinonasal microbiome. Prior research has demonstrated that immunosuppression and antimicrobial exposure can reduce microbial diversity and promote colonization by pathogenic organisms, which perpetuates chronic inflammation and impedes mucosal healing.^19^, ^20^

The incidence rate of de novo CRS was similar between kidney (2.3%) and liver (2.4%) transplant recipients in this study, suggesting that the development of CRS may reflect a broader immunologic or transplant‐related susceptibility rather than an organ‐specific risk. Additionally, transplant rejection and additional organ transplant remained substantial independent risk factors for CRS after adjusting for asthma and allergy, which may be indicative of a cumulative immunosuppressive burden or systemic inflammatory responses.^3^ The need for increased immunosuppression following rejection episodes is a well‐established principle in transplant medicine and is reflected in standard protocols for both kidney and liver transplantation.^11^, ^21^ Similarly, the need to prevent rejection of multiple allografts, the increased immunologic risk from prior sensitization, and the additive effect of repeated induction and maintenance regimens all contribute to a higher cumulative immunosuppressive dose in recipients of additional organ transplants.^11^, ^22^ These observations are consistent with the established concept of a “net state of immunosuppression,” in which the cumulative impact of immunosuppressive therapy, host factors, and environmental exposures collectively determines the risk of infections and immune‐mediated complications across all solid organ transplant recipients.^23^

Histopathologic studies have suggested that CRS in immunosuppressed patients has a unique inflammatory profile.^24^ In our cohort, nearly all patients (34/35, 97.1%) who developed de novo CRS had CRSsNP, a phenotype commonly associated with neutrophilic inflammation and type 1 and 3 inflammatory endotypes. Although inflammatory endotype was not directly measured histopathologically in this cohort, the predominance of CRSsNP aligns with endotypic patterns previously described in immunosuppressed patients. This interpretation is further supported by prior studies demonstrating reduced eosinophilia and increased neutrophilic infiltration in sinonasal tissue from transplant recipients.^16^ In contrast, CRSwNP is more typically associated with impaired fibrinolysis, excessive fibrin deposition, and type 2 eosinophilic inflammation.^25^ Additionally, all transplant recipients in our study were treated with calcineurin inhibitors, which have been shown to reduce eosinophil counts and type 2 cytokines (interleukin [IL]‐4, IL‐5, and IL‐13) in allergic and eosinophilic disease models.^26^ This immunomodulatory effect may contribute to a shift toward type 1 and type 3, neutrophilic‐dominant CRS endotypes in this population. Such a shift has important clinical implications considering the growing body of evidence supporting an association between the presence of neutrophilic inflammation in CRS with a more severe disease course and recalcitrance to medical therapies such as corticosteroids and biologics.^27^, ^28^, ^29^

Prior studies evaluating CRS in transplant recipients have grouped together heterogeneous transplant populations, including both solid and non‐solid organ recipients, despite important differences in comorbidities and immunosuppressive regimens.^8^ This approach limits the generalizability of their findings to specific transplant populations. Our study helps address these gaps by providing organ‐specific incidence estimates within a large, well‐defined cohort from the largest integrated transplant provider in the United States and by excluding all patients with pre‐existing sinonasal complaints, allowing for a more accurate assessment of true de novo CRS. This work also contributes to the literature by describing CRS phenotype distribution and surgical intervention rates in kidney and liver transplant recipients.

This study has several limitations. First, its retrospective design limits the ability to establish causality and introduces the potential for incomplete documentation. However, the use of a large, centralized electronic medical record system across multiple Mayo Clinic sites helps minimize missing data and supports the reliability of diagnostic coding by board‐certified otolaryngologists. Second, the relatively short follow‐up period may underestimate the true incidence of de novo CRS over time. Still, a two‐year follow‐up window captures the early post‐transplant period, when immunosuppression is most intense and risk of immune‐related complications is highest, likely identifying the majority of clinically significant CRS cases. Third, there is potential for detection bias, as transplant recipients undergo more frequent clinical evaluations and imaging compared to the general population. While this may increase CRS detection, it also reflects real‐world clinical surveillance and highlights the relevance of CRS as a complication in this closely monitored population. Additionally, although we excluded patients with documented sinonasal complaints prior to transplantation, it remains possible that some patients had pre‐existing symptoms but did not seek care within our system. Nonetheless, excluding patients with prior sinonasal visits represents the best currently available method for identifying truly de novo CRS in a retrospective cohort. Finally, while kidney and liver transplant recipients typically receive similar core immunosuppressive therapies, the liver's unique immunologic properties may permit slight variation in dosing or drug selection. We attempted to address this by presenting incidence rates separately for each organ group, which revealed remarkably similar CRS incidence between kidney and liver recipients.

Results from our study reflect preliminary findings. Future research, including prospective, multicenter studies with longer follow‐up periods, is needed to confirm our findings and assess long‐term CRS outcomes such as disease progression, quality of life, and treatment responsiveness. Furthermore, future studies investigating the impact of specific immunosuppressive regimens on the development of CRS are crucial to better understand the potential risks associated with these essential therapies. Finally, evaluating whether earlier ENT consultation reduces CRS‐related morbidity among patients who undergo kidney or liver transplantation may help optimize postoperative care pathways and support early recognition and treatment, with the goal of improving quality of life in this vulnerable and growing patient population.

## Conclusion

Kidney and liver transplant recipients experience a higher incidence of de novo CRS compared to the general population. Transplant rejection and multiple organ transplantation significantly increase this risk. Clinicians should maintain a high index of suspicion for CRS in liver and kidney transplant recipients, particularly those with complex transplant courses, to support timely diagnosis and management in this vulnerable population.

## Author Contributions

**Bastien A. Valencia‐Sanchez**, methodology, software, formal analysis, investigation, writing—original draft, writing—review and editing, visualization; **Hannah Daniel**, Investigation, data curation, writing—review and editing; **Prishae Wilson:** investigation, data curation, writing—original draft; **Natasha Najmi**, investigation, data curation, writing—original draft; **Ryan Goodman**, investigation, writing—original draft; **Hani Wadei**, Conceptualization, resources, writing—review and editing, supervision; **Denise Harnois**, conceptualization, resources, writing—review and editing, supervision; **Angela M. Donaldson**, conceptualization, methodology, resources, writing—review and editing, supervision.

## Disclosures

### Competing interests

None.

### Funding source

None.
